# Generalization of a Deep Learning Model for Continuous Glucose Monitoring–Based Hypoglycemia Prediction: Algorithm Development and Validation Study

**DOI:** 10.2196/56909

**Published:** 2024-05-24

**Authors:** Jian Shao, Ying Pan, Wei-Bin Kou, Huyi Feng, Yu Zhao, Kaixin Zhou, Shao Zhong

**Affiliations:** 1 Guangzhou Laboratory, Guangzhou, China; 2 Department of Endocrinology, Kunshan Hospital Affiliated to Jiangsu University, Kunshan, China; 3 Department of Electrical and Electronic Engineering, The University of Hong Kong, Hong Kong, China; 4 Chongqing Fifth People’s Hospital, Chongqing, China

**Keywords:** hypoglycemia prediction, hypoglycemia, hypoglycemic, blood sugar, prediction, predictive, deep learning, generalization, machine learning, glucose, diabetes, continuous glucose monitoring, type 1 diabetes, type 2 diabetes, LSTM, long short-term memory

## Abstract

**Background:**

Predicting hypoglycemia while maintaining a low false alarm rate is a challenge for the wide adoption of continuous glucose monitoring (CGM) devices in diabetes management. One small study suggested that a deep learning model based on the long short-term memory (LSTM) network had better performance in hypoglycemia prediction than traditional machine learning algorithms in European patients with type 1 diabetes. However, given that many well-recognized deep learning models perform poorly outside the training setting, it remains unclear whether the LSTM model could be generalized to different populations or patients with other diabetes subtypes.

**Objective:**

The aim of this study was to validate LSTM hypoglycemia prediction models in more diverse populations and across a wide spectrum of patients with different subtypes of diabetes.

**Methods:**

We assembled two large data sets of patients with type 1 and type 2 diabetes. The primary data set including CGM data from 192 Chinese patients with diabetes was used to develop the LSTM, support vector machine (SVM), and random forest (RF) models for hypoglycemia prediction with a prediction horizon of 30 minutes. Hypoglycemia was categorized into mild (glucose=54-70 mg/dL) and severe (glucose<54 mg/dL) levels. The validation data set of 427 patients of European-American ancestry in the United States was used to validate the models and examine their generalizations. The predictive performance of the models was evaluated according to the sensitivity, specificity, and area under the receiver operating characteristic curve (AUC).

**Results:**

For the difficult-to-predict mild hypoglycemia events, the LSTM model consistently achieved AUC values greater than 97% in the primary data set, with a less than 3% AUC reduction in the validation data set, indicating that the model was robust and generalizable across populations. AUC values above 93% were also achieved when the LSTM model was applied to both type 1 and type 2 diabetes in the validation data set, further strengthening the generalizability of the model. Under different satisfactory levels of sensitivity for mild and severe hypoglycemia prediction, the LSTM model achieved higher specificity than the SVM and RF models, thereby reducing false alarms.

**Conclusions:**

Our results demonstrate that the LSTM model is robust for hypoglycemia prediction and is generalizable across populations or diabetes subtypes. Given its additional advantage of false-alarm reduction, the LSTM model is a strong candidate to be widely implemented in future CGM devices for hypoglycemia prediction.

## Introduction

Diabetes is a serious long-term disease with considerable influence on global health [1]. Type 1 diabetes mellitus (T1DM) is a disease in which the pancreas produces little or no insulin [2], whereas insulin resistance and insufficient insulin are the primary contributors to the development of type 2 diabetes mellitus (T2DM) [3]. Although the pathogenic mechanisms of T1DM and T2DM are different, glucose-lowering treatments such as insulin administration are the common leading cause of hypoglycemia events in patients with both diabetes subtypes [4]. Severe hypoglycemia is a frequent phenomenon in patients with T1DM, with an annual prevalence of 30%-40% [5]. Although the risk of severe hypoglycemia in patients with T2DM is relatively lower, 46%-58% of these patients were reported to have experienced mild hypoglycemia symptoms over a 6-month period [6]. Patients experiencing frequent hypoglycemia events have 1.5-6.0 times increased risks of cardiovascular events and mortality than those without such events [7]. Patients with T2DM from Southeast Asia appear to have an elevated risk of hypoglycemia, as these patients are more often treated with a premixed insulin formulation, are younger, and have a lower BMI than those of their counterparts from Western countries [8-11]. Given that demographic and clinical factors such as ethnic group, diabetes subtype, and BMI are all important components of the complex risk profile of hypoglycemia, accurate risk prediction and prevention of hypoglycemia across populations and diabetes types remain significant challenges in diabetes management.

Recently, continuous glucose monitoring (CGM) has demonstrated good potential to predict hypoglycemia. For patients who wear insulin pumps or those who require multiple daily insulin injections, hypoglycemia prediction based on CGM data could provide a timely warning of impending hypoglycemia for the individual to take immediate action and increase their glucose levels. CGM devices are designed to produce time-series data by recording interstitial glucose concentrations within a relatively short interval of 5-15 minutes over a few days. Therefore, it is possible to leverage the early glucose readings to predict hypoglycemia events over the short-to-medium time horizon. Time-series forecast algorithms such as autoregressive and moving-average algorithms were first adopted to utilize the short-term temporal features of CGM data to predict hypoglycemia [12-15]. A small study including 17 patients with T1DM showed that these CGM-based algorithms achieved 86% sensitivity but only 58% specificity in hypoglycemia prediction [16]. Similar results from studies implementing these time-series forecast algorithms indicated that the low specificity might frequently generate false alarms, leading to discontinuation of CGM use in hypoglycemia prevention [17,18].

To improve the sensitivity and particularly the specificity of hypoglycemia prediction, both traditional machine learning algorithms such as support vector machine (SVM) and random forest (RF) models, along with deep learning models such as the convolutional neural network and long short-term memory network (LSTM) have been used to leverage more temporal features of CGM data [19-25]. When the features, including the mean of glucose and range of time in hyperglycemia, based on CGM data collected over the previous 6 hours were fed into the RF model, hypoglycemia prediction achieved a sensitivity of 93% and a specificity of 91% in a study of 112 patients with T1DM [26]. More recently, when an LSTM deep learning model was implemented on CGM data for hypoglycemia prediction, it achieved a sensitivity of 97% with remarkably few false alarms (0.9 false alarms per week) on a test data set including 10 patients with T1DM, thereby illuminating a path toward the widespread clinical adoption of CGM in hypoglycemia prediction [27].

However, a well-known challenge in implementing predictive models is their generalization [28]. The predictive performance of models could be substantially reduced when used in a setting that is not well-represented by the training data set [29,30]. This is particularly relevant in the case of hypoglycemia prediction, as the previously developed models for this purpose were mostly trained on a small data set of patients with T1DM from Western populations. In addition, the lack of a common test data set rendered the comparison of predictive performances between models unreliable. With recent improvements in measurement accuracy, CGM devices have also gained momentum and have begun to be adopted more widely for the management of T2DM, including in developing countries. Therefore, the established hypoglycemia prediction models should be validated in more diverse populations and over a wide spectrum of patients with different types of diabetes.

We hypothesized that the promising LSTM model for hypoglycemia prediction from CGM data could maintain good predictive performance in different settings for different populations. In this study, we assembled two large CGM data sets from China and the United States, both including patients with T1DM and patients with T2DM. We developed the LSTM model on the Chinese data set and then examined the model performance in the data set from European-Americans in the United States. Apart from exploring the model’s generalization ability for T1DM and T2DM separately, we also compared the predictive performance of the LSTM model with that of SVM and RF models to further indicate its translational potential.

## Methods

### Ethical Considerations

The study protocol was approved by the ethics committees of Kunshan Hospital Affiliated to Jiangsu University (2023-03-014-H01-K01) and the study was performed in accordance with the principles of the Declaration of Helsinki. Written informed consent was obtained from each participant before taking the measurements. The data analyzed were anonymized. All participants volunteered to participate in the project with no compensation provided.

### Data Collection

We collected a primary data set comprising 1578 days of CGM data collected from 264 Chinese people with diabetes to develop a deep learning model for hypoglycemia prediction. The individuals’ glucose levels were monitored using the Medtronic MiniMed CGM device, which requires calibration according to self-monitored blood glucose levels. This CGM device can record glucose levels every 5 minutes over 3 days.

The mean absolute relative difference (MARD) was used to evaluate the quality of the CGM data. The MARD represents the average of the absolute error between all CGM values and matched reference values. A small MARD indicates that the CGM readings are close to the reference glucose value, whereas a larger MARD percentage indicates greater discrepancies between the CGM and reference glucose values. Each individual had at least 5 self-monitoring of blood glucose (SMBG) measurements. As reference glucose values, the SMBG was used to calculate the MARD of CGM data. The data for 72 participants were filtered out because their MARD was higher than 15%, leaving data for 192 participants with 808 days of CGM data for analysis.

**Figure 1.**
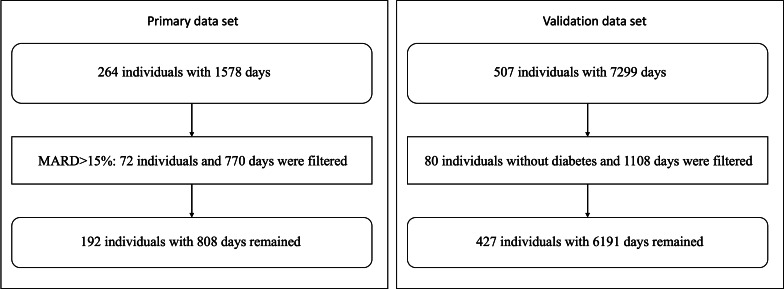
Flowchart of exclusion criteria for the primary data set and validation data set. MARD: mean absolute relative difference.

To examine whether the deep learning model trained and developed with data from the Chinese population could be generalized to a different population, we assembled a large validation data set that mainly comprised data from individuals of European-American ancestry. The validation data set shared by the A1c-Derived Average Glucose study group includes 507 participants and 7299 days of CGM data, also collected with Medtronic MiniMed devices [31]. After filtering out individuals without diabetes, 427 patients with either T1DM or T2DM were included to validate the model. This validation data set was split into two groups: the T1DM group of 268 participants with 3932 days of CGM data and the T2DM group of 159 participants with 2259 days of CGM data. Figure 1 provides the flowchart of exclusion criteria for the primary data set and validation data set.

### Outcome

The glucose values reported by CGM devices were classified into three categories: nonhypoglycemic level (glucose>70 mg/dL), mild hypoglycemic level (glucose=54-70 mg/dL), and severe hypoglycemic level (glucose<54 mg/dL) according to the international consensus on CGM utility [32].

### Data Preprocessing

The primary data set consisting of 192 patients was randomly split into three disjoint data sets, namely the training data set, development data set, and test data set, at a 7:1.5:1.5 ratio. The training data set was used to train the model, whereas the development data set was used to select the hyperparameters in the training process. The test data set was used to evaluate the performance of the developed model.

The CGM sensor may fail to detect a valid glucose level, resulting in the CGM device missing glucose values continuously. To preserve as much of the CGM data as possible, we divided an individual’s CGM data into different segments at the time points of missing data rather than discarding all of the CGM data. A segment was removed if it was shorter than 6 hours (72 data points). We set each glucose value reported by the CGM device as a predictive target if there were sufficient data prior to the target time at which the predictive target was located. The data used to predict the hypoglycemic level of the predictive target were retrieved from a 6-hour time window spanning from −390 minutes to −30 minutes of the target time. After preprocessing the primary data set, the training, development, and test data sets included 100,879, 21,895, and 21,324 samples generated from 134, 29, and 29 participants, respectively. Similarly, the T1DM group and T2DM group from the validation data set contained 712,018 and 405,224 samples generated from 268 and 159 participants, respectively.

### Model Development

We used the common bidirectional LSTM model containing both forward and backward layers to capture the long-range temporal features in the time-series CGM data and to combine these features with context factors [33]. Each LSTM layer consists of 128 memory cells [34]. We chose a set of context factors, including gender, age, diabetes type, and hemoglobin A_1c_ value, to capture the background risk of hypoglycemia and enhance the model’s predictive performance [26]. Therefore, each input data sample included 72 points of CGM data collected during 6 hours and the context factors. The output was the probability of the target glucose value being at the nonhypoglycemic level, mild hypoglycemic level, and severe hypoglycemic level.

We trained the LSTM model to predict the categories of a CGM value within 30 minutes on the prediction horizon. The training process would be terminated if the accuracy failed to increase for 10 consecutive epochs. We used root mean square propagation [35] as the optimizer and set the mini batch size to 64. The LSTM model was developed using the Python package Keras [36]. We also developed models to implement the SVM and RF algorithms for comparison. The SVM model was developed using the radial basis function as the kernel function, which was also used in previous studies of hypoglycemia prediction [37]. The RF model included 100 trees and was developed with the Scikit-learn Python package [38] under default parameters. The input to the SVM and RF models was the same as that used for the LSTM model.

### Model Evaluation

Sensitivity, specificity, and the area under the receiver operating characteristic curve (AUC) were used to evaluate model performance. The label for each sample was the category of a single CGM data point. Sensitivity and specificity indicate the proportion of the labels of CGM data points that were correctly predicted. The DeLong method was used to measure the 95% CIs for the AUC values [39]. All methods of evaluation were developed using Python and the pROC R package [40].

## Results

### Characteristics of the Data Set

Table 1 summarizes the characteristics of the primary data set and the validation data set. As expected, the average age of patients with T1DM was lower than that of the patients with T2DM in both data sets (Wilcoxon rank sum test, *P*<.001).

**Table 1.** Characteristics of the primary data set and validation data set.

Variables		Primary data set		Validation data set	
		Type 2 diabetes (n=175)	Type 1 diabetes (n=17)	Type 2 diabetes (n=159)	Type 1 diabetes (n=268)
Age (years), mean (SD)		53.30 (11.78)	40.59 (13.02)	55.64 (9.32)	43.06 (12.85)
Women, n (%)		51 (29.14)	11 (64.71)	81 (50.94)	140 (52.24)
**Predictive targets, n**					
	Nonhypoglycemia	129,609	12,029	396,415	660,111
	Mild hypoglycemia	1350	336	5985	28,287
	Severe hypoglycemia	608	166	2824	23,620
Hemoglobin A_1c_ (%), mean (SD)		7.69 (1.71)	8.46 (2.22)	7.01 (1.24)	7.51 (1.30)

### Model Performance on the Primary Test Data Set

Using the primary data set from 192 individuals, the three models of LSTM, SVM, and RF were trained and we then evaluated their performance based on the AUC. At the mild hypoglycemic level, the LSTM model achieved an AUC of 97.22% (95% CI 96.78%-97.66%), which was significantly higher than the AUC of 94.33% (95% CI 93.13%-95.53%) and 94.81% (95% CI 93.72%-95.91%) achieved by the SVM and RF models, respectively (both *P*<.001). At the severe hypoglycemic level, the LSTM model achieved an AUC of 99.64% (95% CI 99.53%-99.76%), which was significantly higher than the AUC of 98.30% (95% CI 98.00%-98.60%) and 97.88% (95% CI 96.93%-98.83%) achieved by the SVM and RF models, respectively (both *P*<.001). These results demonstrated that the LSTM model could outperform the SVM and RF models in predicting hypoglycemia.

### Model Generalization on the Validation Data Set

We then utilized the validation data set from 427 European-Americans to evaluate the generalization of the LSTM model developed from our primary data set of 192 Chinese individuals. The LSTM model achieved an AUC of 94.61% (95% CI 94.51%-94.71%) for mild hypoglycemia, which was significantly higher than the AUC of 92.59% (95% CI 92.48%-92.71%) and 91.43% (95% CI 91.28%-91.58%) achieved by the SVM and RF models, respectively (both *P*<.001). The LSTM model achieved an AUC of 96.40% (95% CI 96.25%-96.55%) for severe hypoglycemia, which was significantly higher than the AUC of 95.27% (95% CI 95.15%-95.39%) and 95.17% (95% CI 95.01%-95.32%) achieved by SVM and RF models, respectively (both *P*<.001). Although AUC values of the LSTM model decreased by approximately 3% in the validation data set compared to those from the primary test data set, the overall AUC was still higher than 94%, indicating that the LSTM model could accurately predict hypoglycemia in a different population.

Next, the generalizability of the LSTM model to various disease subtypes was evaluated in the subgroups of T1DM and T2DM from the validation data set. For T1DM, the LSTM model achieved an AUC of 93.49% (95% CI 93.38%-93.61%) at the mild hypoglycemia level, which was significantly higher than the AUC of 90.92% (95% CI 90.78%-91.06%) and 89.74% (95% CI 89.57%-89.92%) achieved by the SVM and RF models, respectively (both *P*<.001). In addition, the LSTM model achieved an AUC of 95.89% (95% CI 95.73%-96.05%) at the severe hypoglycemia level, which was significantly higher than the AUC of 94.06% (95% CI 93.91%-94.21%) and 94.53% (95% CI 94.37%-94.70%) achieved by the SVM and RF models, respectively (both *P*<.001).

For T2DM, the LSTM model achieved an AUC of 96.83% (95% CI 96.66%-97.01%) at the mild hypoglycemia level, which was significantly higher than the AUC of 95.72% (95% CI 95.51%-95.93%) and 94.08% (95% CI 93.73%-94.43%) achieved by the SVM and RF models, respectively (both *P*<.001). In addition, the LSTM model achieved an AUC of 97.65% (95% CI 97.27%-98.04%) at the severe hypoglycemia level, which was significantly higher than the AUC of 96.02% (95% CI 95.70%-96.34%) and 95.71% (95% CI 95.23%-96.19%) achieved by the SVM and RF models, respectively (both *P*<.001).

The AUCs of the LSTM model were consistently higher than those from the SVM and RF models in both the T1DM and T2DM data sets. Taken together, these results demonstrated that the LSTM model could be generalized to different diabetes subtypes without significant loss of predictive performance.

### Comparison of the False Alarm Rate

Finally, we examined whether the LSTM model could achieve a low false alarm rate (ie, high specificity) under satisfactory sensitivity. According to previous studies of hypoglycemia prediction, we set the model parameters to fix the satisfactory sensitivity level at 90% and 95% for mild and severe hypoglycemia prediction, respectively [21,26,37]. As shown in Table 2, while maintaining a sensitivity of 90% for mild hypoglycemia, which is difficult to predict, the LSTM model could achieve a specificity of 88.43%, which was higher than the specificity obtained from the SVM and RF models. For severe hypoglycemia, when a higher satisfactory sensitivity rate of 95% was set, the LSTM model achieved a specificity of 87.34%, which was higher than that obtained from the SVM model. Moreover, the RF model could not achieve a sensitivity of 95% for the severe hypoglycemic level. Taken together, these results demonstrated that the LSTM model could maintain a lower false alarm rate than the SVM and RF models in clinically practical settings.

**Table 2.** Specificity and sensitivity of the three models on the validation data set.

	Mild hypoglycemic level		Severe hypoglycemic level	
	Specificity (%)	Sensitivity (%)	Specificity (%)	Sensitivity (%)
LSTM^a^	88.43	90.00	87.34	95.00
SVM^b^	82.57	90.00	80.67	95.00
RF^c^	82.65	90.00	Not determined	Not achieved

a LSTM: long short-term memory.

b SVM: support vector machine.

c RF: random forest.

## Discussion

### Principal Findings

In this study, we assembled two large CGM data sets from China and the United States to develop and validate an LSTM deep learning model for hypoglycemia prediction. The LSTM model could maintain good predictive performance when applied to data sets from a different ethnic population or any common subtype of diabetes. The LSTM model could also predict both mild and severe hypoglycemia with higher accuracy than the traditional SVM and RF models. While targeting clinically meaningful high sensitivity, the LSTM model could achieve high specificity, thereby reducing the rate of false alarms.

Compared with the models tested without external validation in most previous studies of hypoglycemia prediction, we developed an LSTM model and validated the model in a data set from a different population to examine its generalizability [27]. There are considerable differences in dietary structure and clinical practice between China and the United States, which are among the many factors that might affect the risk of hypoglycemia. Previous studies demonstrated that clinical models trained in one population could result in an AUC reduction as great as 15% when applied to a distinct population [41-43]. However, the LSTM model derived from our Chinese training data set maintained high prediction performance (AUC>93%) with only a minor loss of 3% in the US data set, indicating good generalizability of the model. As CGM devices are becoming more widely adopted, the generalizability of the LSTM model could be further improved by training the model with data from multiple populations or can be fine-tuned for the target population using a transfer-learning approach [44].

We also examined the generalizability of the LSTM model on another dimension of diabetes pathogenicity. Given the different pathogenic mechanisms between T1DM and T2DM, hypoglycemia occurring in different diabetes subtypes would be expected to be preceded by various patterns of glucose fluctuation, which could be leveraged by the LSTM model for prediction. Therefore, the model was expected to lose predictive performance when the training and validation data sets had different proportions of diabetes subtypes. Indeed, we observed a higher AUC value for T2DM than for T1DM in the validation data set, which was likely due to the fact that our training data set primarily consisted of individuals with T2DM. However, for either subtype of diabetes, the LSTM model consistently maintained an AUC value above 93%, indicating the good generalizability of the model. With the increasing popularity of CGM usage in the management of all subtypes of diabetes, the LSTM model could be further improved by using larger training data sets with a wider representation of the various diabetes subtypes.

Achieving high sensitivity has been the main focus of previous models for hypoglycemia prediction, as severe hypoglycemia requires immediate external intervention [15,32]. With the sacrifice of high specificity, false alarms became an obstacle for the safe and widespread use of CGM devices [45-47]. False-alarm fatigue could lead to users ignoring the true alarms of hypoglycemia and contribute to the discontinuation of CGM use [45]. Moreover, glucose control could be compromised, as CGM users may frequently take action to elevate their glucose level when a false alarm is generated [46]. Therefore, it is imperative to balance the false alarm rate with sufficient sensitivity of the prediction. In this study, we demonstrated that the LSTM model would generate fewer false alarms than the traditional machine learning models under satisfactory sensitivity rates of 90% and 95% for mild and severe hypoglycemia, respectively. Therefore, the balanced hypoglycemia prediction performance from the LSTM model demonstrated that it has potential to promote the use of CGM in a variety of clinical settings.

One reason for the better predictive performance of the LSTM model than the SVM and RF models might be that the LSTM algorithm is more suitable for analyzing sequential data. CGM data are a type of sequential data that are generated in time order. The LSTM algorithm consists of memory cells that learn the sequential nature of observations within CGM data [48]. The input of one memory cell is the glucose value taken at one time point and then the LSTM takes all of the glucose values as inputs sequentially. Every memory cell retains the relevant information and discards irrelevant information for the predictive task, and then the relevant information in one cell is delivered to the next cell [49-53]. With this sequential structure, LSTM networks incorporate CGM data from the past to accurately make predictions of hypoglycemia risk in the near future.

### Limitations

There are several limitations of this study. Although we tested the generalizability of the LSTM model using two data sets from China and the United States, further validation might still be required for application of the model in other countries. Similarly, as only T1DM and T2DM were included in our data sets, the model should be tested with wider and more representative training data sets to validate its utility on other minority subtypes of diabetes. Moreover, data from only one CGM device manufacturer were available for this study. Thus, it is unknown whether the model would perform equally well with data collected from other devices such as factory-calibrated CGM or noninvasive CGM devices. However, given that all of the devices were strictly calibrated by finger-stick glucose values, the fluctuation patterns and temporal dependence of CGM data, which are key factors for the LSTM prediction task, should be largely captured by any certified CGM device. Moreover, the performance of the LSTM model for hypoglycemia prediction will need to be further validated in a CGM data set without missing data.

### Conclusions

We developed an accurate LSTM model for mild and severe hypoglycemia prediction using a large data set of 619 patients with diabetes from China and the United States. The model could be robustly generalized to different populations or any common subtype of diabetes. Moreover, while maintaining satisfactory levels of sensitivity, the model could also achieve high specificity, indicating its potential to mitigate the hypoglycemia false-alarm fatigue that is frequently observed in clinical practice. Taken together, we demonstrated that the LSTM model is a strong candidate algorithm to be further tested and implemented for the wider clinical adoption of CGM.

## Abbreviations

AUC: area under the receiver operating characteristic curve
CGM: continuous glucose monitoring
LSTM: long short-term memory
MARD: mean absolute relative difference
RF: random forest
SMBG: self-monitoring of blood glucose
SVM: support vector machine
T1DM: type 1 diabetes mellitus
T2DM: type 2 diabetes mellitus

## References

[1] Deshpande AD, Harris-Hayes M, Schootman M (2008). Epidemiology of diabetes and diabetes-related complications. Phys Ther.

[2] Atkinson MA, Eisenbarth GS, Michels AW (2014). Type 1 diabetes. Lancet.

[3] Chatterjee S, Khunti K, Davies MJ (2017). Type 2 diabetes. Lancet.

[4] Cryer PE (2008). The barrier of hypoglycemia in diabetes. Diabetes.

[5] Frier BM (2009). The incidence and impact of hypoglycemia in type 1 and type 2 diabetes. Inter Diab Monitor.

[6] Silbert R, Salcido-Montenegro A, Rodriguez-Gutierrez R, Katabi A, McCoy RG (2018). Hypoglycemia among patients with type 2 diabetes: epidemiology, risk factors, and prevention strategies. Curr Diab Rep.

[7] International Hypoglycaemia Study Group (2019). Hypoglycaemia, cardiovascular disease, and mortality in diabetes: epidemiology, pathogenesis, and management. Lancet Diabetes Endocrinol.

[8] Chan JCN, Malik V, Jia W (2009). Diabetes in Asia: epidemiology, risk factors, and pathophysiology. JAMA.

[9] Kalra S, Balhara YPS, Sahay BK, Ganapathy B, Das AK (2013). Why is premixed insulin the preferred insulin? Novel answers to a decade-old question. J Assoc Physicians India.

[10] Goh SY, Hussein Z, Rudijanto A (2017). Review of insulin-associated hypoglycemia and its impact on the management of diabetes in Southeast Asian countries. J Diabetes Investig.

[11] Aschner P, Sethi B, Gomez-Peralta F (2015). Insulin glargine compared with premixed insulin for management of insulin-naïve type 2 diabetes patients uncontrolled on oral antidiabetic drugs: the open-label, randomized GALAPAGOS study. J Diabetes Complications.

[12] Eren-Oruklu M, Cinar A, Quinn L, Smith D (2009). Estimation of future glucose concentrations with subject-specific recursive linear models. Diabetes Technol Ther.

[13] Yang J, Li L, Shi Y, Xie X (2019). An ARIMA model with adaptive orders for predicting blood glucose concentrations and Hypoglycemia. IEEE J Biomed Health Inform.

[14] Eren-Oruklu M, Cinar A, Rollins DK, Quinn L (2012). Adaptive system identification for estimating future glucose concentrations and hypoglycemia alarms. Automatica (Oxf).

[15] Dassau E, Cameron F, Lee H (2010). Real-time hypoglycemia prediction suite using continuous glucose monitoring: a safety net for the artificial pancreas. Diabetes Care.

[16] Bayrak ES, Turksoy K, Cinar A, Quinn L, Littlejohn E, Rollins D (2013). Hypoglycemia early alarm systems based on recursive autoregressive partial least squares models. J Diabetes Sci Technol.

[17] Tansey M, Laffel L, Cheng J (2011). Satisfaction with continuous glucose monitoring in adults and youths with type 1 diabetes. Diabet Med.

[18] Ramchandani N, Arya S, Ten S, Bhandari S (2011). Real-life utilization of real-time continuous glucose monitoring: the complete picture. J Diabetes Sci Technol.

[19] Georga EI, Protopappas VC, Ardigò D, Polyzos D, Fotiadis DI (2013). A glucose model based on support vector regression for the prediction of hypoglycemic events under free-living conditions. Diabetes Technol Ther.

[20] Jensen MH, Christensen TF, Tarnow L, Seto E, Dencker Johansen M, Hejlesen OK (2013). Real-time hypoglycemia detection from continuous glucose monitoring data of subjects with type 1 diabetes. Diabetes Technol Ther.

[21] Mosquera-Lopez C, Dodier R, Tyler NS (2020). Predicting and preventing nocturnal hypoglycemia in type 1 diabetes using big data analytics and decision theoretic analysis. Diabetes Technol Ther.

[22] Gu W, Zhou Z, Zhou Y, He M, Zou H, Zhang L Predicting blood glucose dynamics with multi-time-series deep learning.

[23] Chen J, Li K, Herrero P, Zhu T, Georgiou P Dilated recurrent neural network for short-time prediction of glucose concentration.

[24] Doike T, Hayashi K, Arata S, Mohammad KN, Kobayashi A, Niitsu K A blood glucose level prediction system using machine learning based on recurrent neural network for Hypoglycemia prevention.

[25] Li J, Ma X, Tobore I (2020). A novel CGM metric-gradient and combining mean sensor glucose enable to improve the prediction of nocturnal hypoglycemic events in patients with diabetes. J Diabetes Res.

[26] Dave D, DeSalvo DJ, Haridas B (2021). Feature-based machine learning model for real-time hypoglycemia prediction. J Diabetes Sci Technol.

[27] Mosquera-Lopez C, Dodier R, Tyler N, Resalat N, Jacobs P (2019). Leveraging a big dataset to develop a recurrent neural network to predict adverse glycemic events in type 1 diabetes. IEEE J Biomed Health Inform.

[28] Zhang Y, Wu H, Liu H, Tong L, Wang MD (2019). Improve model generalization and robustness to dataset bias with bias-regularized learning and domain-guided augmentation. arXiv.

[29] Kortylewski A, Egger B, Schneider A, Gerig T, Morel-Forster A, Vetter T Analyzing and reducing the damage of dataset bias to face recognition with synthetic data. https://ieeexplore.ieee.org/xpl/mostRecentIssue.jsp?punumber=8972688.

[30] Tian Y, Chen W, Zhou T, Li J, Ding K, Li J (2020). Establishment and evaluation of a multicenter collaborative prediction model construction framework supporting model generalization and continuous improvement: a pilot study. Int J Med Inform.

[31] Nathan DM, Kuenen J, Borg R (2008). Translating the A1C assay into estimated average glucose values. Diabetes Care.

[32] Danne T, Nimri R, Battelino T (2017). International consensus on use of continuous glucose monitoring. Diabetes Care.

[33] Gers FA, Schmidhuber J, Cummins F (2000). Learning to forget: continual prediction with LSTM. Neural Comput.

[34] Hochreiter S, Schmidhuber J (1997). Long short-term memory. Neural Comput.

[35] Hinton G, Srivastava N, Swersky K Neural networks for machine learning. Lecture 6a. Overview of mini-batch gradient descent. Computer Science University of Toronto.

[36] Keras.

[37] Oviedo S, Contreras I, Quirós C, Giménez M, Conget I, Vehi J (2019). Risk-based postprandial hypoglycemia forecasting using supervised learning. Int J Med Inform.

[38] Pedregosa F, Varoquaux G, Gramfort A (2011). Scikit-learn: machine learning in python. J Mach Learn Res.

[39] DeLong ER, DeLong DM, Clarke-Pearson DL (1988). Comparing the areas under two or more correlated receiver operating characteristic curves: a nonparametric approach. Biometrics.

[40] Robin X, Turck N, Hainard A (2011). pROC: an open-source package for R and S+ to analyze and compare ROC curves. BMC Bioinformatics.

[41] Lemeshow S, Teres D, Klar J, Avrunin JS, Gehlbach SH, Rapoport J (1993). Mortality probability models (MPM II) based on an international cohort of intensive care unit patients. JAMA.

[42] Adrie C, Francais A, Alvarez-Gonzalez A (2009). Model for predicting short-term mortality of severe sepsis. Crit Care.

[43] Riley RD, Ensor J, Snell KIE (2016). External validation of clinical prediction models using big datasets from e-health records or IPD meta-analysis: opportunities and challenges. BMJ.

[44] Torrey L, Shavlik J (2010). Handbook of Research on Machine Learning Applications and Trends: Algorithms, Methods, and Techniques.

[45] Shivers JP, Mackowiak L, Anhalt H, Zisser H (2013). “Turn it off!”: diabetes device alarm fatigue considerations for the present and the future. J Diabetes Sci Technol.

[46] Cryer PE (2014). Glycemic goals in diabetes: trade-off between glycemic control and iatrogenic hypoglycemia. Diabetes.

[47] Wong JC, Foster NC, Maahs DM (2014). Real-time continuous glucose monitoring among participants in the T1D Exchange clinic registry. Diabetes Care.

[48] Kong W, Dong ZY, Jia Y, Hill DJ, Xu Y, Zhang Y (2017). Short-term residential load forecasting based on LSTM recurrent neural network. IEEE Trans Smart Grid.

[49] Xu Z, Li S, Deng W Learning temporal features using LSTM-CNN architecture for face anti-spoofing.

[50] Shi X, Jin Y, Dou Q, Heng P-A (2020). LRTD: long-range temporal dependency based active learning for surgical workflow recognition. Int J Comput Assist Radiol Surg.

[51] Liao J, Liu L, Duan H (2022). Using a convolutional neural network and convolutional long short-term memory to automatically detect aneurysms on 2D digital subtraction angiography images: framework development and validation. JMIR Med Inform.

[52] Athanasiou M, Fragkozidis G, Zarkogianni K, Nikita KS (2023). Long short-term memory–based prediction of the spread of influenza-like illness leveraging surveillance, weather, and Twitter data: model development and validation. J Med Internet Res.

[53] Ayyoubzadeh SM, Ayyoubzadeh SM, Zahedi H, Ahmadi M, R Niakan Kalhori S (2020). Predicting COVID-19 incidence through analysis of Google trends data in Iran: data mining and deep learning pilot study. JMIR Public Health Surveill.

